# Supplementary material for: Inadvertent QRS prolongation by an optimization device-based algorithm in patients with cardiac resynchronization therapy
Source: PLoS One. 2022 Sep 26;17(9):e0275276. doi: 10.1371/journal.pone.0275276 (PMC9512171; doi:10.1371/journal.pone.0275276)
Supplement: Attachment — Submitted filename: Response to Reviewers.docx [file pone.0275276.s001.docx]

Response to reviewers

We would like to thank both reviewers of our manuscript. We acknowledge and appreciate their careful reading of the manuscript as well as their expert analysis of its strengths and potential weak points. We address herein all their comments and hope they will find our response satisfactory and the revised manuscript worth publishing.

**Response to Reviewer #1**

**Comment 1.** Although repeated measures ANOVA would be the natural approach here, dependent-data pairwise t-test comparisons are acceptable. However, both t-tests and ANOVA methods require normality of the data. Either the authors are capable to provide evidence of normality of the measurements by a formal test or they should repeat the analysis by nonparametric methods that don't require the normality assumption.

Response:

We tested the normality with the use of Shapiro-Wilk’s W test. This was done for all three QRSd differences and QuickOpt-suggested VVD. The results are as follows:

1) Delta QRSd (native versus CRT with VVD=0): P = 0.09

2) Delta QRSd (native versus CRT with QuickOpt-VVD): P = 0.19

3) Delta QRSd (CRT with VVD=0 versus CRT with QuickOpt-VVD): P = 0.17

4) QuickOpt-suggested VVD: P = 0.46

Therefore, the hypothesis of normality for all distributions should not be rejected.

Changes in the manuscript:

The information on normality testing was included in the “Statistical Analysis” paragraph as follows: ***“Differences in pairs of QRSd were tested for normality of distribution (Shapiro-Wilk’s W test) and compared by the Student´s t-test for dependent samples.”***

The presentation of the results remained unchanged.

**Comment 2.** The statistical analysis assumes a homogeneous sample. However, Table 1 displays heterogeneous characteristics of the observed subjects. Again, I see two options here. Either the authors argue that these characteristics are not confounding factors. Or, pairwise comparisons should be adjusted for these characteristics, e.g. using a linear mixed model for repeated measures, where the information of Table 1 is included as a battery of covariates.

Response:

Only a few baseline (clinical and CRT-related) characteristics are confounding factors. Among them, QuickOpt-proposed VVD was already mentioned in the former version of the manuscript. Now, we included detailed univariate and multivariate regression analysis with baseline characteristics as independent variables and CRT effects (QRS shortening with VVD=0 and QRSd prolongation with QuickOpt-proposed VVD) as dependent variables. This analysis does not change the main message of the study. Besides the well-known association between native QRSd duration and CRT effects, it highlights again that the QuickOpt-proposed VVD is the main and independent determinant of the adverse impact of QuickOpt-based optimization.

Changes in the manuscript:

We have completely rewritten the paragraph „Impact of VVD on QRS widening“. Now, its title reads ***„Impact of baseline characteristics on CRT effects“****,* and the text was modified as follows:

***„Table 2 shows the results of univariate and multivariate linear regression analysis with baseline (clinical and CRT-related) characteristics as independent variables and CRT effects (QRS shortening with VVD=0 and QRSd prolongation with QuickOpt-proposed VVD) as dependent variables. Besides the well-known association between native QRSd duration and CRT effects, this analysis highlights that the QuickOpt-proposed VVD is the main and independent determinant of the adverse impact of QuickOpt-based optimization. This is also illustrated in Figure 4 demonstrating that the relative QRSd prolongation due to the QuickOpt programming correlates positively with the absolute value of VVD (R = 0.66, P <0.00001).”***

The sentence regarding the regression analysis was included in the „Statistical Analysis” paragraph as follows: ***„Associations of baseline factors and the CRT effects were assessed by univariable and multivariable linear regression with a forward stepwise selection method.”***

**Response to Reviewer #2**

Reviewer´s comments:

The concept of evaluating the real effect of automated QRS optimization (QuickOpt in this case) is very interesting. The demonstrated QRS prolongation after optimization is important finding.
It should be noted that while shortening of the QRS was associated with improved outcomes in some studies, the reasons for wide QRS could be different and not always related to conduction system dysfunction. Also, pacing the site with latest intrinsic activation may not necessarily lead to better outcomes.

Response: We agree with all said above.

In order to better evaluate the article the following should be addressed:

1. Do we have any clinical outcomes?

Response:

We acknowledge the instinct of physicians to connect physiological experiments with outcomes. This study, however, was never intended to be a clinical outcome study but rather a technical evaluation protocol for a given device algorithm. We believe that such studies should become a standard evaluation tool of such technologies and precede their entry into the phase of clinical outcome trials, since they may (as in this case) uncover flawed designs and prevent potential damage inflicted by direct clinical testing. In summary: we, unfortunately, have no systematic (and publishable) follow-up data for this cohort since it was never planned.

In response to comments from reviewer #1, we included in the manuscript detailed univariate and multivariate regression analysis with baseline characteristics as independent variables and CRT effects as dependent variables (please see a new Table 2). This analysis addresses the majority of your concerns below:

2. Do we have data stratified by ischemic versus nonischemic CMP?

Response:

The etiology of cardiomyopathy was not associated with CRT effects (because of the small sample).

3. Do we have data stratified by the LV lead position?

Response:

LV lead position was not associated with CRT effects (because of the small sample).

4. How QRS optimization affected patients with initially more narrow versus more wide QRS, same about narrow versus wide VDD=0?

Response:

The impact of native QRS duration on the outcome of QuickOpt-based optimization was rather weak. On the contrary, QuickOpt-proposed VVD was the main and independent determinant of the adverse impact of QuickOpt-based optimization.

5. Did optimization improve those patients, who had wider VVD=0 than intrinsic QRS (I counted at least 7) and who they were (type of conduction, etiology, lead position, etc)?

Response:

There were 7 patients in whom CRT with VVD=0 programming prolonged the QRSd. In six of them, the QuickOpt-based optimization resulted in further prolongation of QRSd. In one patient, the QRSd was prolonged by 4 ms (with VVD=0) and shortened by 5 ms (with QuickOpt-VVD), i.e. with a net benefit of 1 ms compared to the native QRSd. No change in the manuscript was done.

6. Consider recalculate statistics for LBBB only (without IVCD) patients.

Response:

The presence of true LBBB was associated with CRT effects in the case of VVD=0 but this factor was eliminated in multivariate analysis because of covariance with the significant factor of QRSd. On the contrary, the presence of true LBBB was not associated with the impact of QuickOpt optimization. Just for your information, in the subgroup of patients with true LBBB (n=30), native QRSd was shortened by 23 ms by VVD=0 programming and by 11 ms by QuickOpt-guided VVD. Net harm caused by QuickOpt (QRSd prolongation by 12 ms) was the same as in the total sample. We do not consider this information essential. It was not included in the manuscript.
