# Supplementary material for: Inadvertent QRS prolongation by an optimization device-based algorithm in patients with cardiac resynchronization therapy
Source: PLoS One. 2022 Sep 26;17(9):e0275276. doi: 10.1371/journal.pone.0275276 (PMC9512171; doi:10.1371/journal.pone.0275276)
Supplement: Attachment — Submitted filename: Inadvertent QRS prolongation by an optimization device-based algorithm in patients with cardiac resynchronization therapy.docx [file pone.0275276.s002.docx]

**"Inadvertent QRS prolongation by an optimization device-based algorithm in patients with cardiac resynchronization therapy"**

**Review**

**COMMENT**

**Evaluation of resynchronization optimization through manual versus automatic method based on device algorithms is a very interesting idea, since there is vast literature that evidences clinical benefits in the narrowing of QRS complex.**

**CONSIDERATIONS**

**1-Do we have clinical outcomes?**

**2- Is there a relationship between the degree of QRS reduction and coronary sinus electrode position?**

**3-Is there a difference in the degree of narrowing QRS in regarding to gender?**

**4-Do we have echocardiogram outcomes and degree of narrowing QRS complex?**
