# Supplementary material for: Inadvertent QRS prolongation by an optimization device-based algorithm in patients with cardiac resynchronization therapy
Source: PLoS One. 2022 Sep 26;17(9):e0275276. doi: 10.1371/journal.pone.0275276 (PMC9512171; doi:10.1371/journal.pone.0275276)
Supplement: Attachment — Submitted filename: Response to Reviewers.docx [file pone.0275276.s003.docx]

Response to reviewers

We would like to thank the reviewers of our manuscript. We acknowledge and appreciate their contribution to improvement of the manuscript and are glad that our responses satisfied their queries.

Despite the fact, that all reviewers stated that we were able to adequately address all issues raised in the previous review round, following additional comments were submitted by the reviewer #4. We appreciate his concerns and respond to them below. We also refer to our previous response to reviewers that included answers to similar or identical comments.

**Response to Reviewer #4**

**Comment 1 and 4.**

Do we have clinical (and echocardiographic) outcomes?

Response:

We have addressed this issue in our previous response to reviewers: “We acknowledge the instinct of physicians to connect physiological experiments with outcomes. This study, however, was never intended to be a clinical outcome study but rather a technical evaluation protocol for a given device algorithm. We believe that such studies should become a standard evaluation tool of such technologies and precede their entry into the phase of clinical outcome trials, since they may (as in this case) uncover flawed designs and prevent potential damage inflicted by direct clinical testing. In summary: we, unfortunately, have no systematic (and publishable) follow-up data for this cohort since it was never planned.”

**Comment 2.** Is there a relationship between the degree of QRS reduction and coronary sinus electrode position?

Response:

This comment was also addressed in our previous response to reviewers: “LV lead position was not associated with CRT effects (because of the small sample).”

**Comment 3.** Is there a difference in the degree of narrowing QRS in regarding to gender?

Response: There was no difference in QRS change induced by CRT between the male and female participants of the study (11.0 ± 10.3 vs. 13.4 ± 13.6 ms, P = 0.6 for the t-test statistics). Since this analysis was not prespecified and has no relevant context within the manuscript, we prefer not to include it.

Changes made in the manuscript: none
